# Class-modeling analysis reveals T-cell homeostasis disturbances involved in loss of immune control in elite controllers

**DOI:** 10.1186/s12916-018-1026-6

**Published:** 2018-02-28

**Authors:** José M. Benito, María C. Ortiz, Agathe León, Luis A. Sarabia, José M. Ligos, María Montoya, Marcial Garcia, Ezequiel Ruiz-Mateos, Rosario Palacios, Alfonso Cabello, Clara Restrepo, Carmen Rodriguez, Jorge del Romero, Manuel Leal, María A. Muñoz-Fernández, José Alcamí, Felipe García, Miguel Górgolas, Norma Rallón, On behalf of ECRIS integrated in the Spanish AIDS Research Network

**Affiliations:** 1 IIS-Fundación Jiménez Díaz, UAM, Av. Reyes Católicos, 2, 28040 Madrid, Spain; 2 Hospital Universitario Rey Juan Carlos, Móstoles, Madrid Spain; 3 Universidad de Burgos, Burgos, Spain; 4 Hospital Clinic-IDIBAPS, HIVACAT, Universidad de Barcelona, Barcelona, Spain; 5 Centro Nacional de Investigaciones Cardiovasculares, Madrid, Spain; 6 Hospital Virgen del Rocío, Sevilla, Spain; 7 Unidad de E. Infecciosas. Hospital Virgen de la Victoria e IBIMA, Málaga, Spain; 8 Hospital Universitario Fundación Jiménez Díaz, Madrid, Spain; 9 Centro Sanitario Sandoval, Instituto de Investigación Sanitaria del Hospital Clínico San Carlos (IdISSC), Madrid, Spain; 10 Laboratory of Molecular Immuno-Biology, Hospital General Universitario Gregorio Marañón, Instituto de Investigación Sanitaria Gregorio Marañón, Madrid, Spain; 11 AIDS Immunopathology Unit, Instituto de Salud Carlos III, Majadahonda, Madrid Spain

**Keywords:** Elite controllers, CD4 T-cell loss, Class modeling, T-cell homeostatic parameters, CD8 exhaustion

## Abstract

**Background:**

Despite long-lasting HIV replication control, a significant proportion of elite controller (EC) patients may experience CD4 T-cell loss. Discovering perturbations in immunological parameters could help our understanding of the mechanisms that may be operating in those patients experiencing loss of immunological control.

**Methods:**

A case–control study was performed to evaluate if alterations in different T-cell homeostatic parameters can predict CD4 T-cell loss in ECs by comparing data from EC patients showing significant CD4 decline (cases) and EC patients showing stable CD4 counts (controls). The partial least-squares–class modeling (PLS-CM) statistical methodology was employed to discriminate between the two groups of patients, and as a predictive model.

**Results:**

Herein, we show that among T-cell homeostatic alterations, lower levels of naïve and recent thymic emigrant subsets of CD8 cells and higher levels of effector and senescent subsets of CD8 cells as well as higher levels of exhaustion of CD4 cells, measured prior to CD4 T-cell loss, predict the loss of immunological control.

**Conclusions:**

These data indicate that the parameters of T-cell homeostasis may identify those EC patients with a higher proclivity to CD4 T-cell loss. Our results may open new avenues for understanding the mechanisms underlying immunological progression despite HIV replication control, and eventually, for finding a functional cure through immune-based clinical trials.

**Electronic supplementary material:**

The online version of this article (10.1186/s12916-018-1026-6) contains supplementary material, which is available to authorized users.

## Background

Human immunodeficiency virus-1 (HIV-1) infection is mostly characterized by ongoing viral replication leading to a progressive decline of CD4+ T cells and eventually progression to clinical AIDS. Nevertheless, there is a small subset of HIV-1+ subjects who are able to spontaneously control HIV-RNA replication in plasma to levels below the limit of detection in the absence of antiretroviral therapy. They are called elite controllers (ECs) and represent less than 1% of the total of HIV-infected patients [1]. ECs have been considered as a model of spontaneous functional cure and the role of host genetic factors, innate and adaptive immune responses, viral characteristics, and their interactions have been analyzed in several studies [1–5].

However, ECs constitute a heterogeneous group in terms of genetic and immunologic characteristics [6], and no single mechanism has been described as being responsible for controlling viral replication [5–7]. These patients are also heterogeneous from the clinical point of view, since some of them show either virologic and/or immunologic progression [8–11]. Studies with large cohorts of EC patients have reported that as many as 25% of them experience virological progression (losing the ability to maintain HIV replication control) [11], and around 20% experience immunological progression (a decline in CD4 counts) even in the absence of virological progression [8, 9], as we have also previously demonstrated in our cohort of HIV controllers of the Spanish AIDS Research Network (the ECRIS cohort) [11]. However, the mechanisms accounting for this CD4 T-cell loss in ECs remain largely unknown.

Given the relevance of different alterations of T-cell homeostasis in the CD4 depletion observed in HIV patients with uncontrolled viral replication [12–14], some of these same mechanisms might be operating in EC patients experiencing CD4 T-cell loss. In this regard, previous studies have shown that even though EC patients are able to suppress HIV replication to an undetectable level, they present different T-cell homeostasis disturbances compared to uninfected controls and/or to HIV non-controllers with suppression of HIV replication mediated by combination antiretroviral therapy (cART) [15–20], suggesting that T-cell homeostasis disturbances are still operating in EC patients and could potentially be involved in the immunological progression.

To test this hypothesis, and with the final aim of finding predictors of immunological progression, herein we have comprehensively compared several important parameters of T-cell homeostasis in EC patients with long-lasting HIV replication control but experiencing immunological progression with those for EC patients showing no progression. The partial least-squares–class modeling (PLS-CM) methodology was applied as a statistical modeling technique to discriminate between the two groups of patients, and as a predictive model.

## Methods

### Study population

This is a case–control study including adult patients with chronic HIV infection and with long-term spontaneous control of HIV replication (EC patients) during the whole study follow-up period, recruited from the cohort of HIV controllers of the Spanish AIDS Research Network (ECRIS cohort) launched in 2013. ECRIS is an open multicenter cohort of HIV controller patients. The data are from the Spanish Long-Term Non-Progressors (LTNP) cohort and the Spanish AIDS Research Network (CoRIS) cohort [21], and from different clinical centers in Spain. To be included in the ECRIS cohort, EC patients were defined as asymptomatic individuals with at least three consecutive plasma HIV viral load (pVL) determinations below the detection limit (pVL < 50 copies/mL) during at least 12 months of follow-up, in the absence of any cART. The characteristics of the ECRIS cohort have been described in detail elsewhere [11]. Only those EC subjects maintaining long-term control of HIV replication (more than 3 years) with regular immunovirological follow-ups (CD4 counts and pVL) and with available baseline cryopreserved cellular samples were included in the study. Baseline was the beginning of the follow-up period as an EC patient.

### Selection of EC study groups

**Fig. 1:**
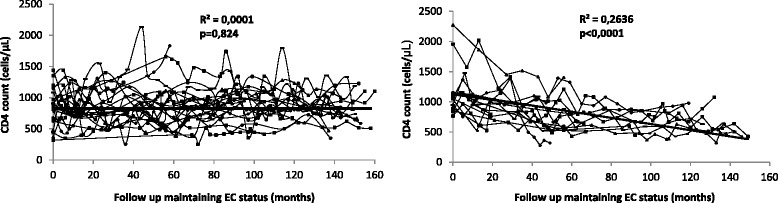
Scatterplots showing the evolution of the CD4 count during the follow-up period for two groups of EC patients: those showing stable CD4 counts (left) and those showing a significant CD4 decline (right). Each individual line represents data from one patient. EC elite controller

The relevant CD4 data for each patient were measured during the follow-up period if the patient maintained their EC status. All of these data were used in a linear regression analysis to estimate the CD4 slope for the patient. According to the CD4 slope, the study population was categorized into two groups: EC controls, patients with stable CD4 counts during the follow-up period (CD4 count slope not significantly different from zero) and EC cases, patients whose CD4 counts declined during the follow-up period (a statistically significant and negative CD4 count slope). A total of 36 EC patients were included: 22 being EC controls and 14 being EC cases (Additional file 1 and Fig. 1). Moreover, 14 age- and sex-matched healthy controls (HIV-uninfected individuals) were included as a healthy control group (HCs).

### Cell samples

Anonymized samples from both groups of patients were kindly provided by the HIV BioBank, which is integrated into the Spanish AIDS Research Network (RIS) [22]. Anonymized data were taken from the ECRIS cohort database (see Additional file 1), according to the sample assignment agreement reference 19/15 dated 25 August 2015. All analyses were done with cryopreserved peripheral blood mononuclear cells (PBMCs), and were performed when both groups of EC patients (cases and controls) presented similar levels of CD4 counts, that is prior to CD4 T-cell loss in EC cases. EDTA-anticoagulated blood was obtained by venipuncture. PBMCs were immediately isolated by density gradient centrifugation using Ficoll-Hypaque (Sigma Chemical Co., St. Louis, MO), frozen in fetal calf serum plus 10% dimethyl sulfoxide, and stored in liquid nitrogen in the Spanish AIDS Research Network HIV BioBank. The viability of thawed PBMCs was always greater than 85%.

### Immunophenotypic analysis

We used a comprehensive approach of simultaneously measuring different T-cell parameters involved in HIV pathogenesis: differentiation stage; recent thymic emigrants (RTEs); activation, exhaustion, and apoptosis of T memory stem cells (Tscm cells); T regulatory cells (Treg cells); replicative senescence; and response to homeostatic cytokines. We selected a total of 16 different monoclonal antibodies distributed in two different panels of 11 and 13 antibodies (plus a cell viability marker), which in combination defined 350 different unique T-cell subsets that were analyzed by multiparametric flow cytometry. A complete list of all monoclonal antibodies and fluorochromes used in the study as well as antibodies included in each of the two staining panels is shown in Additional file 2. A minimum of 2 million of PBMCs from each patient were stained with each antibody panel, and a detailed description of staining conditions and definitions of T-cell subsets analyzed is given in Additional file 3.

### Statistical analyses

The main characteristics of the study population and the different parameters evaluated are expressed as median and interquartile range. Comparisons between groups were done using a Mann–Whitney *U* test. The two-tailed *p* values were considered as significant only when they were lower than 0.05. All statistical analyses were performed using the SPSS software version 15 (SPSS Inc., Chicago, IL, USA). PLS-CM was employed to discriminate between the different groups of subjects included in the study (control patients, case patients, and HCs). Two PLS models were generated: one to discriminate between EC patients and HCs (PLS-1) and another to discriminate between cases and control groups of EC patients (PLS-2). In these models, 350 immunological parameters were used as predictor (explicative) variables of the model, and the subjects’ group was the predicted (response) binary variable. For PLS-1, a value of 0 was assigned to EC patients and a value of 1 was assigned to HCs. For PLS-2, a value of 0 was assigned to EC controls and a value of 1 to EC cases.

In the first step, PLS was used to select a minimum set of predictor variable to reduce the original 350 immunological variables to simplify the analysis without losing any information. The parameter employed to make this selection, called *variable influence on projection* (VIP), summarizes the importance of the predictor variable on the model taking into account the amount of explained variance of the predicted (response) variable.

In the second step, a PLS model was built using the selected set of predictor variables grouped together to generate a minimum set of latent variables (LVs) that are not directly observed (measured) but rather inferred (through a mathematical model) from other variables that are observed (directly measured). Each LV is a linear combination of the original set of observable variables. A clear advantage of using LVs is that it reduces the dimensionality of data. The number of LVs included in the PLS model is optimized to maximize the explained variance of the *X* (predictor variables) and *Y* (response variable) components of the model. Applying this PLS model, a predicted value for the response variable was estimated for each sample and for these predicted values, a probability distribution was generated. For this distribution, the probabilities α and ß for type I and II errors of the hypothesis test, respectively, were calculated. The sensitivity [(1- α) × 100] and specificity [(1- ß) × 100] of the PLS model were used to assign each sample to its correct group. A detailed explanation of PLS-CM is given in Additional file 4.

## Results

### Patient characteristics

The CD4 slope during the follow-up period for controls and cases is shown in Table 1. Of the 36 EC patients included in the study, 22 were considered controls (stable CD4 counts during the follow-up period) and 14 were considered cases (significant CD4 decline during the follow-up period). All immunological parameters were measured prior to CD4 T-cell loss in EC cases. The evolution of CD4 counts during the follow-up period for controls and cases is shown in Fig. 1. The main characteristics of the controls and cases are shown in Table 1. There were no significant differences between control and case groups in terms of age, median time since diagnosis, CD4 count at the beginning of follow-up, CD4 count at the commencement of the study, percentage of patients infected with hepatitis C virus, percentage of patients presenting with non-AIDS defining events during the follow-up period, and number of HIV-RNA blips during the follow-up period.

**Table 1:** Characteristics of patients included in the study

Characteristic	Control group (*n* = 22)	Case group (*n* = 14)	*p* value
Age (years)^a^	40 [35, 48]	42 [39, 48]	0.58
Gender (% of males)	33	69	**0.04**
Follow-up maintaining EC status (years)	12 [10, 13]	9 [5, 12]	**0.02**
Time since HIV diagnosis (years)	19.3 [14.8, 24.1]	19.3 [9.6, 21.8]	0.62
CD4 count at the beginning of follow-up (cells/μL)	838 [646, 1097]	1047 [881, 1125]	0.06
CD4 count at the end of follow-up (cells/μL)	931 [675, 1147]	632 [443, 843]	**0.01**
CD4 slope (cells/μL per year)	8.9 [1.2, 19]	-66 [− 113, −32]	**< 0.0001**
CD4 count at the moment of the study (cells/μL)	889 [752, 1051]	886 [548, 1280]	0.87
Hepatitis C virus positive (%)	80	69	0.68
Patients with non-AIDS defining events during the follow-up period (%)	11	11	1
Number of HIV-RNA blips (pVL > 50 copies/mL)	2 [0, 2.3]	1 [0, 2.3]	0.43

*EC* elite controller

^a^Data for continuous variables are given as median [interquartile range]

### PLS class modeling of EC patients versus HC subjects (PLS-1)

**Fig. 2:**
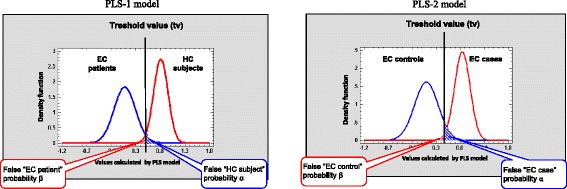
Density functions for values calculated by models PLS-1 (left) and PLS-2 (right) for the two classes of individuals: EC patients and HC subjects for the PLS-1 model, and EC cases and EC controls for the PLS-2 model. The vertical line represents a threshold value chosen to differentiate between the classes. The small overlapping areas to the right and to the left of this line represent the probabilities α and β, respectively. EC elite controller, HC healthy control

First a PLS model was generated to discriminate between EC patients and HIV-seronegative HC subjects. A data matrix with 50 samples (36 EC patients and 14 HCs) and 350 variables was used to fit a first PLS model without any data preprocessing. A binary variable took the value 0 if the sample was from an EC patient and 1 if it was from a HC. This model let us choose 93 variables with VIP values higher than 1 so that we could simplify the analysis without losing any information. A new PLS model (PLS-1) with the same criteria and a reduced data matrix with 93 variables was built. Three LVs were included in the final PLS model, and the percentage of explained variance of the response variable (*y*) and predictor variables (*X*) is shown in Table 2 for PLS-1. A normal distribution for each class (EC patients and HCs) was fitted with the values of the response variable calculated by the PLS model because the *p* values of the normality tests were greater than 0.1. These density functions for each class are plotted in Fig. 2. The mean and standard deviation of the calculated response variable for the PLS-1 model for the EC patient and HC classes are, respectively, 0.075 ± 0.219 and 0.810 ± 0.146 (Fig. 2).

**Table 2:** Percentage of variance of predictor (*X* block) and response (*y* block) variables explained by the PLS models. Individual (partial) and accumulated (total) explained variance for each latent variable of the models are shown

Latent variable	PLS-1				Latent variable	PLS-2			
	*X* block		*y* block			*X* block		*y* block	
	Partial	Total	Partial	Total		Partial	Total	Partial	Total
1	91.4	91.4	31.6	31.6	1	92.1	92.1	38.1	38.1
2	1.4	92.8	43.3	74.9	2	1.4	93.5	26.2	64.3
3	1.1	93.9	6.1	81.0	3	0.8	94.3	16.9	81.2
					4	0.7	95.0	3.4	84.6

Numbers in bold represent the total variance explained by the PLS model

**Fig. 3:**
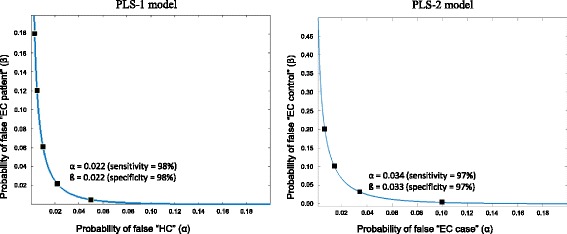
Risk curves ß versus α for the PLS-1 (left) and PLS-2 (right) class models. Different pairs of α and ß are marked as black squares. The pair of equal values of sensitivity and specificity is shown. EC elite controller, HC healthy control

**Fig. 4:**
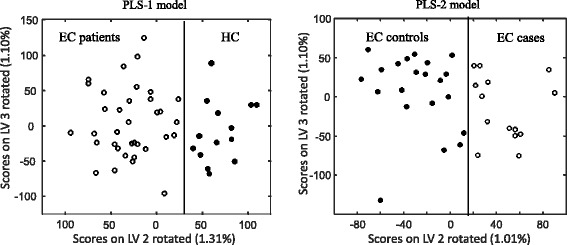
Dot plots showing the projection of the scores for LV2 and LV3 for each subject included in the study. Left: Scores for EC patients (open circles) and HC subjects (black circles) obtained with the PLS-1 model. Right: Scores for EC controls (black circles) and EC cases (open circles) obtained with the PLS-2 model. EC elite controller, HC healthy control, LV latent variable

By choosing a threshold to differentiate between EC patients and HC subjects, we can see in Fig. 2 that the small area under the density function of the class of EC patients on the right of the threshold is the probability α of rejecting the null hypothesis (H_0_: the sample belongs to the EC patient class) when it is true. On the other hand, the small area under the density function of the class of HCs on the left of the threshold is the probability β of accepting the null hypothesis (H_0_) when it is false. By changing the threshold, a risk curve of type α error versus type ß error can be generated and from this we can estimate the pair of equal values of sensitivity [(1-α) × 100] and specificity [(1- ß) × 100] of the model (Fig. 3). For the PLS-1 model, these values were both 98%. To visualize the ability of the PLS model to discriminate between EC patients and HCs, Fig. 4 (left) shows the projection in a plane of the scores for LV2 and LV3 (rotated 25° counterclockwise) for each of the 50 individuals of the study (36 EC patients and 14 HCs). This plot has a vertical line (a rotated LV2 value) that perfectly separates EC patients (on the left) and HC subjects (on the right).

### PLS class modeling of EC cases versus EC controls (PLS-2)

PLS class modeling was applied to discriminate between EC cases (EC patients showing a CD4 decline during the follow-up period) and EC controls (EC patients with stable CD4 counts during the follow-up period). Firstly, by fitting a PLS model with samples from 36 patients (22 EC controls and 14 EC cases), 77 variables from the 350 original ones with a VIP value higher than 1 were selected. No data preprocessing was used and a binary response variable took the value 0 if the sample was from an EC control and the value 1 if it was from an EC case. The same procedure as used for PLS-1 was followed.

A PLS regression (PLS-2) with the same previous criteria and the reduced data matrix with 77 variables was built with four LVs. The percentages of explained variance by the model of the *X* and *y* variables are shown in Table 2 (under PLS-2). We checked that the *p* values of the normality tests were greater than 0.10 and therefore, a normal distribution for each class (EC cases and EC controls) was fitted with the values of the response variable calculated by the PLS model. The normal density functions for each class were plotted (Fig. 2). The mean values and standard deviation of the response variable calculated by the PLS model for the EC case and EC control classes were respectively 0.096 ± 0.247 and 0.846 ± 0.162 (Fig. 2).

By changing the threshold, a risk curve of type α versus type ß errors was generated and from this we estimated the pair of equal values of sensitivity [(1-α) × 100] and specificity [(1- ß) × 100] of the model (Fig. 3). For the PLS-2 model, these values were both 97%. To visualize the ability of the PLS-2 model to discriminate between EC cases and EC controls, Fig. 4 shows the projection in a plane of the scores for LV2 and LV3 rotated 49° counterclockwise. This plot shows has a vertical line (a rotated LV2 value) that perfectly separates EC controls (on the left) and EC cases (on the right).

The PLS model shows that the immunological variables have information with sufficient specificity and sensitivity to discriminate between the EC controls and the EC cases (this is also true for discriminating between EC patients and HC subjects). There is an underlying structure responsible for this capacity of discrimination that is reducible to two LVs. However, the immunological variables are all on the same scale (percentage) and it is not appropriate to normalize them since the variables that are noise in the discrimination acquire the same magnitude as those truly responsible for it and may be incorrectly included in the model. The consequence of not normalizing the data is twofold: (1) one of the LVs is linked to the distance between the vector formed by the mean values ​​of the predictor variables and the origin of coordinates and (2) the immunological variables with a smaller range may not be selected due to a numerical effect. In addition, PLS tends to eliminate highly correlated variables because they do not carry independent information, although their immunological meaning may be relevant and different. Consequently, choosing the immunological variables for which the difference between classes is perceived is done by evaluating the individual distribution of their values, as in the Mann–Whitney *U* test.

### T-cell parameters significantly altered in EC patients with respect to healthy subjects

Of the 93 immunological variables selected by the PLS-1 model to differentiate EC patients from healthy subjects, 43 presented statistically significant differences when EC patients and healthy subjects were compared with the Mann–Whitney test. Since the immunological variables are all on the same scale and it is not appropriate to normalize them, the PLS-CM model did not normalize the variables, and for this reason the immunological variables with a smaller range may not have been selected. Nevertheless, those variables could have some relevant information. Thus, using the Mann–Whitney *U* test, it was possible to evaluate those variables significantly different between EC patients and healthy subjects but that were not selected by the PLS model. In fact, there were another 36 immunological variables significantly different between EC patients and healthy subjects that were not selected by the PLS model due to a numerical effect. A complete list of immunological variables with significant differences is shown in Additional file 5.

**Fig. 5:**
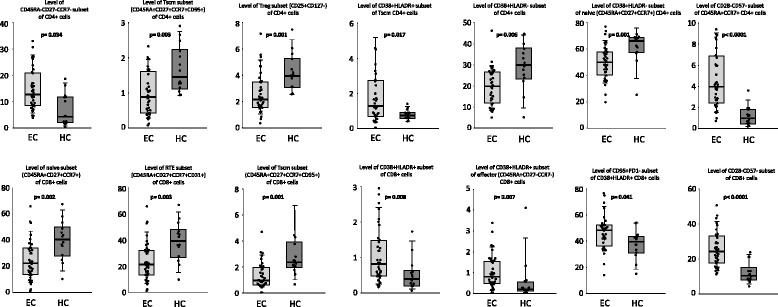
Box plots showing the levels of different CD4 (upper) and CD8 (lower) T-cell subsets in elite controller (EC) patients and in healthy subjects (HC). The *y*-axis represents the percentages of cells. *p* values for the comparison between EC patients and HC (Mann–Whitney *U* test) are shown in the graphs. EC elite controller, HC healthy control

Figure 5 shows levels for the most relevant variables in EC patients and healthy subjects. Among CD4 T-cell subsets, EC patients showed lower levels of Tscm and Treg subsets and higher levels of the CD45RA-CD27-CCR7- subset compared to healthy subjects. Interestingly, expression of activation marker CD38 (but not of HLADR) was also lower in total CD4 T cells and in naïve CD4 T cells of EC patients. In contrast, co-expression of activation markers CD38 and HLADR was higher in Tscm cells of EC patients. The proportion of naïve CD4 cells lacking expression of CD28 was also higher in EC patients.

Among CD8 T-cell subsets, EC patients showed lower levels of naïve RTE (naïve cells expressing CD31) and Tscm subsets compared to healthy subjects. However, co-expression of activation markers CD38 and HLADR in total CD8+ cells and in effector (CD45RA + CD27-CCR7-) subset was higher in EC patients. Expression of CD95 in activated (either CD38+ or HLADR+) subsets of CD8 cells was also higher in EC patients. Lastly, the proportion of CD8 cells lacking CD28 expression was also higher in EC patients (Fig. 5).

### T-cell parameters associated with loss of immunological control in EC patients

Of the 77 immunological variables selected by the PLS model to differentiate between EC cases (EC patients showing a significant CD4 count decline) and EC controls (EC patients with a stable CD4 count), 12 presented statistically significant differences between EC cases and EC controls using the Mann–Whitney test. Interestingly, all of them were variables with the highest coefficients (loadings) in the PLS model. The absolute value of the loading of each immunological variable in the PLS model is a measure of the weight of the variable in the model and thus, the higher the loading the higher the probability of significant differences between the two groups (EC cases and EC controls). In fact, for the entire set of 77 immunological variables selected by the PLS model, there was a significant inverse correlation between the absolute value of the loadings and the *p* values obtained by the Mann–Whitney test (Additional file 6), meaning that the higher the absolute value of the loading, the higher the probability of a statistically significant (*p* < 0.05) difference.

Moreover, using the Mann–Whitney *U* test, it was possible to evaluate those variables significantly different between EC cases and EC controls that were not selected by the PLS model due to a numerical effect. This approach allowed us to detect another nine immunological variables with levels significantly different between EC cases and EC controls. A complete list of immunological variables significantly different by the Mann–Whitney test between EC cases and EC controls is shown in Additional file 7.

**Fig. 6:**
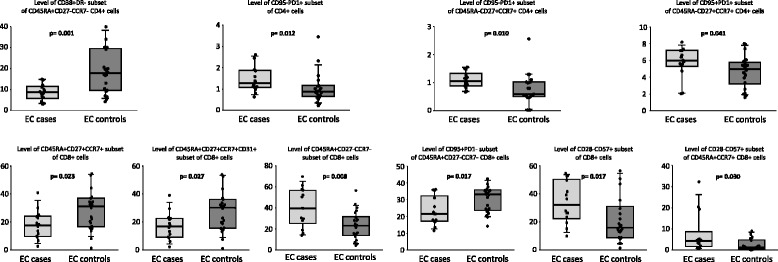
Box plots showing the levels of different CD4 (upper) and CD8 (lower) T-cell subsets in elite controller (EC) patients with a CD4 count decline (EC cases) and in EC patients with a stable CD4 count (EC controls) during the follow-up period. The *y*-axis represents the percentages of cells. *p* values are for the comparison between EC cases and EC controls (Mann–Whitney *U* test). EC elite controller

Figure 6 shows levels for the most relevant variables for EC cases and EC controls. Among CD4 subsets, EC cases presented lower levels of CD38 expression on the effector (CD45RA + CD27-CCR7-) subset of CD4 cells. In contrast, the level of PD1 expression on total and on central memory (CD45RA-CD27 + CCR7+) CD4 cells was higher in EC cases compared to EC controls. Among CD8 T-cell subsets, compared to EC controls, EC cases showed lower levels of naïve (CD45RA + CD27 + CCR7+) and of RTE (naïve cells expressing CD31) subsets. In contrast, the level of the effector (CD45RA + CD27-CCR7-) subset was higher in EC cases as was the level of senescence (CD28-CD57+) in total CD8 cells and in naïve CD8 cells. Lastly, expression of CD95 in the effector subset of CD8 cells was lower in EC cases.

**Fig. 7:**
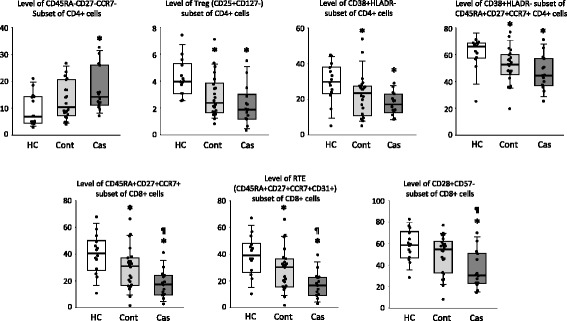
Box plots showing the levels of different CD4 (upper) and CD8 (lower) T-cell subsets in healthy subjects (HCs), elite controller (EC) patients with a stable CD4 count (Cont), and in EC patients with a CD4 count decline (Cas) during the follow-up period. The *y*-axis represents the percentages of cells. Significant differences (*p* < 0.05) with respect to HCs are marked with an asterisk (*) and between the Cas and Cont groups of EC patients with the ¶ symbol. EC elite controller, Cas EC case, Cont EC control, HC healthy control

Overall, for those T-cell subsets that were significantly different in EC patients with respect to HCs, the levels for EC controls were intermediate between those found in EC cases and HCs (Fig. 7). Thus, levels in EC controls were more similar to levels in HCs than were levels in EC cases, and for some of these subsets, the levels for EC controls and HCs were not significantly different. This was the case for levels of the CD45RA-CD27-CCR7- subset of CD4 cells and for the levels of CD28 + CD57- subset of CD8 cells (Fig. 7).

## Discussion

The present study was designed to test the hypothesis that perturbations in immunological parameters related to T-cell homeostasis may be associated with CD4 T-cell loss in EC patients maintaining long-term undetectable levels of plasma HIV viremia. The main findings of our study are: (1) there is an important perturbation of T-cell homeostasis in EC patients despite long-term undetectable viremia; (2) based on the T-cell parameters analyzed, a PLS-CM statistical approach was able to discriminate and to predict, with high sensitivity and specificity, EC patients experiencing immunological progression from those EC patients with a stable CD4 count; and (3) immunological parameters associated with the loss of CD4 T cells in EC patients included mainly those related to CD8 T-cell homeostasis.

Despite the persistent control of HIV replication to undetectable levels, several alterations related to a state of systemic inflammation have been recognized in ECs compared to seronegative subjects and to HIV patients with cART-mediated control of HIV replication [23–25], and this has been associated with an increased rate of morbidity and mortality in EC patients [24–26]. Our study demonstrates that immunological alterations in EC patients are not limited to markers of systemic inflammation and extend to several cellular markers of T-cell homeostasis. Although the EC patients included in our study had maintained long-term undetectable plasma HIV viremia, they clearly departed from seronegative healthy subjects (HCs) regarding the distribution of T-cell subsets. As many as 93 immunological variables were necessary to discriminate between EC patients and HCs and many of these presented levels significantly different between ECs and HCs, suggesting there is an important perturbation of T-cell homeostasis in EC patients despite long-term undetectable viremia.

Different processes involved in T-cell homeostasis were altered in ECs compared to healthy subjects. Among them, naïve and RTE subsets, stem cell memory subsets, regulatory T cells, and expression of the costimulatory receptor CD28 were significantly decreased, whereas activation and expression of CD95 were significantly increased. Some of these perturbations have already been reported in different cohorts of ECs, such as alterations in naïve/memory subsets [17, 19], increased activation [15, 18, 27], increased senescence [27], and decreased regulatory T cells [28]. However, EC cohorts employed in these previous studies had varying degrees of HIV-associated immune suppression (as reflected in CD4 counts) and different cutoffs of plasma viremia, meaning that some alterations reported in these studies could be influenced by CD4 counts [28] or low-level plasma HIV viremia [27]. Since all EC patients included in our study had high CD4 counts at the time of the immunophenotypic analysis, our results demonstrate that perturbation of T-cell homeostasis is already present in ECs from the early stages of HIV disease. Moreover, our study is the first reporting alterations in ECs of some important parameters of T-cell homeostasis such as Tscm cells [29, 30]. Interestingly Tscm cells were diminished in both CD4 and CD8 T cells and thus, the potential to repopulate other subsets of memory cells [31] may be compromised in ECs at early stages of infection.

The most relevant findings of our study are those related to T-cell parameters associated with loss of immunological control in ECs. Using a set of 77 different immunological variables, the PLS model was able to discriminate, with high sensitivity and specificity, between EC patients showing a significant CD4 decline (cases) and those with stable CD4 counts (controls). The immunological variables were assessed when both groups of EC patients presented similar high levels of CD4 counts (prior to CD4 T-cell loss in EC cases) and thus, the differences found cannot be attributed to different degrees of HIV-associated immune suppression, but rather they are characteristic of some EC subjects and likely involved in the decline of CD4 T cells observed in EC cases. Since no functional methods were employed in this study, we can speculate only about the potential mechanisms involved in the differences observed in the extent of T-cell homeostasis disturbance between EC cases and EC controls. Besides viral factors, such as the higher levels of residual viral replication in EC cases [10], several host-related factors could contribute to the CD4 T-cell loss and T-cell homeostasis disturbance observed in EC cases. Among them are defective thymic function [17], lymphoid tissue fibrosis [32], genetic variation in different genes related to immune response [33, 34], and a cytokine-driven inflammatory milieu [35].

Interestingly, the two-sample non-parametric test (Mann–Whitney *U* test) was not able to detect subtle but important differences between cases and controls for many of the immunological variables that were detected by the PLS model. PLS-CM is an approach that is best suited to extracting these differences employing latent factors and building a predictive model in situations with a small sample size and many predictive variables. In agreement with this higher sensitivity of PLS modeling to detect differences among a large panel of variables, the immune parameters that had significant differences by the two-sample test were those showing the highest loadings in the PLS model.

The factors involved in the immunological progression observed in some ECs that maintain virological control are largely unknown and studies addressing this issue are scarce, with only a few investigating potential associated immunological factors [10, 15, 36, 37]. In some of these studies, T-cell activation was shown to be associated with disease progression in ECs using a cross-sectional design [15, 36]. The other two studies addressing the association of T-cell activation with CD4 evolution in EC patients and using a longitudinal follow-up period similar to our study [10, 37] found discordant results. One reported increased activation associated with immunologic progression [10] but the other did not [37]. Differences in the criteria used to define immunological progression may explain the discordant results. Moreover, both studies analyzed T-cell activation when CD4 counts were significantly different between EC patients showing immunological progression and those maintaining stable CD4 counts, which is in clear contrast with our study.

Our results show that several T-cell subsets showed significant differences between EC cases and controls and interestingly they were mainly related to CD8 cell homeostasis. Naïve and RTE subsets of CD8 cells were diminished in EC cases, suggesting either a defect in thymic output or an increased rate of transition of naïve cells to more differentiated subsets. Since a deficit in thymic output would be reflected in the RTE subset of both CD8 and CD4 compartments, our findings more strongly support an increased rate of differentiation. This, together with the finding of increased levels of effector CD8 cells in EC cases, supports the higher levels of residual HIV replication in EC cases. Unfortunately, we did not measure plasma HIV viremia with ultrasensitive assays and thus, we can only speculate on this possibility. However, in support of this hypothesis, previous studies have found higher levels of residual viremia in EC patients showing CD4 depletion [9, 10, 38]. Although levels of effector CD8 cells were higher in EC cases, CD8 T cells showed increased levels of the CD28-CD57+ subset, a phenotype that has been associated with replicative senescence [39]. CD8 T cells expressing this senescent phenotype have lower cytotoxic potential [40, 41] and produce high amounts of inflammatory cytokines such as IL6 and TNFα [39], promoting an inflammatory state that could contribute to the CD4 depletion observed in EC cases. An interesting finding was that naïve CD8 cells of EC cases also have increased levels of senescence, suggesting that they have undergone an antigen-independent expansion without differentiation to memory cells in an attempt to maintain CD8 T-cell homeostasis. Lastly, regarding alterations of CD4 T-cell subsets, EC cases had increased levels of expression of the cell exhaustion-associated marker PD1 in total CD4 cells and in central memory subset. Since overexpression of PD1 has been associated with a lower ability to reconstitute CD4 T cells in HIV patients with cART-mediated suppression of viral replication [42, 43], this increased expression of PD1 in EC cases could be another factor contributing to CD4 depletion.

Finally, there are some caveats that deserve comment. First, studies employing EC patients are very limited in sample size given the very low frequency of this exceptional population of individuals among the whole population of HIV-infected patients. However, in our study, we addressed the potential bias of a small sample size by studying a very homogeneous study population. Also, for the data analyses we employed the PLS regression, which is a statistical technique for performing multivariate calibration, especially when there are more variables than samples. Second, although the objective of our study had no such scope, it could have been interesting to include a group of HIV-normal progressors to assess whether the underlying mechanisms of the loss of immune control in EC patients are comparable to those underlying the non-immunological control in normal HIV+ progressors patients, as well as to evaluate the power of the PLS model to discriminate between both phenomena.

## Conclusions

In summary, the present study is the first to perform an in-depth analysis of T-cell homeostasis disturbances in a well-characterized cohort of ECs with long-term control of HIV replication and showing different evolution of CD4 counts. We used a PLS-CM statistical approach that enabled us to detect subtle but important immunophenotype differences associated with the immunological progression. Overall, our results demonstrate the existence of an important perturbation of T-cell homeostasis in EC patients despite long-term undetectable viremia. For those EC patients experiencing immunological progression, those disturbances are mainly related to CD8 T-cell homeostasis. Our results may open new avenues for understanding the mechanisms underlying immunological progression despite HIV replication control, suggesting that active pathogenic mechanisms are still present in some EC patients. These findings should be considered to improve the clinical management of this exceptional group of patients, and eventually in seeking a functional cure through immune-based clinical trials.

## Additional files

Additional file 1: Flow diagram showing the inclusion criteria and the sequential strategy for selecting the patients included in the study. Numbers inside the boxes indicate the number of patients selected after each step of the selection process. (DOC 200 kb)

Additional file 2: Monoclonal antibodies and fluorochromes used in the study. (DOC 49 kb)

Additional file 3: Staining conditions for immunophenotypic analysis and definitions of T-cell subsets analyzed. (DOC 29 kb)

Additional file 4: Partial least-squares–class modeling (PLS-CM). (DOC 33 kb)

Additional file 5: Immunological variables (in CD4 and CD8 T-cell subsets) with significant differences between EC patients and healthy subjects (HCs) (Mann–Whitney *U* test). (DOC 119 kb)

Additional file 6: Scatterplot showing loading values (coefficients) for the 77 immunological variables selected by the PLS model versus *p* values obtained when comparing levels of these variables between EC cases and EC controls with the Mann–Whitney *U* test. The dotted line marks the threshold for statistical significance (*p* < 0.05). (DOC 67 kb)

Additional file 7: Immunological variables (in CD4 and CD8 T-cell subsets) with significant differences between EC cases and EC controls (Mann–Whitney *U* test). (DOC 52 kb)

Additional file 8: Clinical centers and research groups that contribute to ECRIS. (DOC 33 kb)

## Abbreviations

AIDS: Acquired immune deficiency syndrome
cART: Combination antiretroviral therapy
EC: Elite controller
EDTA: Ethylenediamine tetraacetic acid
HC: Healthy control
HIV-1: Human immunodeficiency virus-1
LTNP: Long-term non-progressor
LV: Latent variable
PBMC: Peripheral blood mononuclear cell
PLS-CM: Partial least-squares–class modeling
pVL: Plasma viral load
RNA: Ribonucleic acid
RTE: Recent thymic emigrant
SPSS: Statistical Package for the Social Sciences
Treg cell: T regulatory cell
Tscm cell: T memory stem cell
VIP: Variable influence on projection
