# Supplementary material for: Class-modeling analysis reveals T-cell homeostasis disturbances involved in loss of immune control in elite controllers
Source: BMC Med. 2018 Feb 28;16:30. doi: 10.1186/s12916-018-1026-6 (PMC5830067; doi:10.1186/s12916-018-1026-6)
Supplement: Supplementary file 1 — Flow diagram showing the inclusion criteria and the sequential strategy for selecting the patients included in the study. Numbers inside the boxes indicate the number of patients selected after each step of the selection process. (DOC 200 kb) [file 12916_2018_1026_MOESM1_ESM.doc]

Additional file 1. Flow diagram showing the inclusion criteria and the sequential strategy of selection of patients included in the study. Numbers inside the boxes indicate the number of patients selected after each step in the selection process.
