# Supplementary material for: Class-modeling analysis reveals T-cell homeostasis disturbances involved in loss of immune control in elite controllers
Source: BMC Med. 2018 Feb 28;16:30. doi: 10.1186/s12916-018-1026-6 (PMC5830067; doi:10.1186/s12916-018-1026-6)
Supplement: Supplementary file 2 — Monoclonal antibodies and fluorochromes used in the study. (DOC 49 kb) [file 12916_2018_1026_MOESM2_ESM.doc]

Additional file 2**.** Monoclonal antibodies and fluorochromes used in the study.

| **Antibody** | **Fluorochrome** | **Clone** | **Provider** | **Staining Panel 1** | **Staining Panel 2** |
| --- | --- | --- | --- | --- | --- |
| CD3 | BV711 | UCHT1 | Biolegend | X | X |
| CD4 | PE-Cy5 | RPA-T4 | Biolegend | X | X |
| CD8 | PerCP-Cy5.5 | RPA-T8 | Biolegend | X | X |
| CD45RA | FITC | HI100 | Biolegend | X | X |
| CD27 | BV570 | O323 | Biolegend | X | - |
| CCR7 | PE-CF594 | 150503 | BD Biosciences | X | X |
| CD31 | BV510 | WM59 | BD Biosciences | X | - |
| CD38 | BV650 | HB-7 | Biolegend | X | X |
| HLA-DR | BV785 | L243 | Biolegend | X | X |
| PD-1 | PE-Cy7 | EH12.2H7 | Biolegend | X | X |
| CD95 | BV605 | DX2 | Biolegend | X | - |
| CD127 | BV605 | A019D5 | Biolegend | - | X |
| CD25 | PE | M-A251 | Biolegend | - | X |
| CD39 | BV421 | A1 | Biolegend | - | X |
| CD28 | BV510 | CD 28.2 | Biolegend | - | X |
| CD57 | BV570 | HNK-1 | Biolegend | - | X |
| - | Live/Dead Violet |  | Molecular Probes | X | X |
