# Supplementary material for: Class-modeling analysis reveals T-cell homeostasis disturbances involved in loss of immune control in elite controllers
Source: BMC Med. 2018 Feb 28;16:30. doi: 10.1186/s12916-018-1026-6 (PMC5830067; doi:10.1186/s12916-018-1026-6)
Supplement: Supplementary file 3 — Staining conditions for immunophenotypic analysis and definitions of T-cell subsets analyzed. (DOC 29 kb) [file 12916_2018_1026_MOESM3_ESM.doc]

Additional file 3. Staining conditions for immunophenotypic analysis and definitions of T-cell subsets analyzed.

Two different antibody panels were used for evaluation of the different CD4 and CD8 T-cell subsets. Combinations of conjugated antibodies in the panels were as follows: Panel 1 included CD3-BV711, CD4-PECy5, CD8-PerCP/Cy5.5, CD45RA-FITC, CD27-BV570, CCR7-PE-CF594, CD38-BV650, HLADR-BV785, PD1-PE/Cy7, CD95-BV605, and CD31-BV510. Panel 2 included CD3-BV711, CD4-PECy5, CD8-PerCP/Cy5.5, CD45RA-FITC, CCR7-PE-CF594, CD38-BV650, HLADR-BV785, PD1-PE/Cy7, CD127-BV605, CD25-PE, CD28-BV510, CD57-BV570 and CD39-BV 421.

Two million of PBMCs were washed with 2 mL of phosphate-buffered saline (PBS) and stained for surface markers by incubation with the appropriate antibody panel for 30 min at 4ºC in the absence of light. Antibodies conjugated with Brilliant Violet were mixed with brilliant stain buffer before being added to the cell samples. After incubation, cells were washed with 2 mL of PBS and resuspended in 150 µL of PBS. Sample acquisition was performed on an SP 6800 Spectral flow cytometer (Sony) and a minimum of 100.000 CD3+CD4+ and CD3+CD8+ cells were acquired for further analysis. Data analysis was performed using FlowJo (Treestar, San Carlos, CA). Gating strategy was as follows: an initial gating was applied using forward (FSC) and side (SSC) scatter, and then FSC area versus FSC height to select single cells. Staining with Live/dead was used to exclude dead cells from further analysis. From the population of single live lymphocytes, a gate was placed to select CD3+CD4+ and CD3+CD8+ T cells. Starting from CD3+CD4+ and CD3+CD8+ cells, the next parameters were analyzed:

**Panel 1**:

1.- Differentiation stage: Using CD45RA, CD27 and CCR7 markers that in combination define 8 different unique subsets including: naïve (CD45RA+CD27+CCR7+), central memory (CD45RA-CD27+CCR7+), transitional memory (CD45RA-CD27+CCR7-), effector memory (CD45RA-CD27-CCR7-), pre-effector (CD45RA+CD27+CCR7-), and effector (CD45RA+CD27-CCR7-), as well as CD45RA+CD27-CCR7+ and CD45RA-CD27-CCR7+ subsets.

2.- Recent thymic emigrants (RTE): Defined as CD45RA+CD27+CCR7+CD31+ cells.

3.- T stem cell memory (Tscm): Defined as CD45RA+CD27+CCR7+CD95+ cells.

4.- Activation: Using CD38 and HLA-DR as surrogate markers of activation. Three different subsets were considered: CD38+DR-, CD38+DR+ and CD38-DR+. The level of activation was evaluated in the different subsets defined by CD45RA, CD27 and CCR7, as well as in RTE and Tscm subsets.

5.- Exhaustion and apoptosis: Using PD1 as a surrogate marker of exhaustion and CD95 (Fas Ligand) as a surrogate marker of apoptosis. Levels of PD1 and/or CD95 were evaluated in the different subsets defined by CD45RA, CD27 and CCR7, as well as in RTE and Tscm subsets.

**Panel 2**:

1.- Differentiation stage: Using CD45RA and CCR7 markers that in combination define 4 different unique subsets including: naïve (CD45RA+CCR7+), central memory (CD45RA-CCR7+), effector memory (CD45RA-CCR7-), and effector (CD45RA+CCR7-) subsets.

2.- T regulatory (Treg) cells: Defined as CD25+CD127- cells. Expression of CD39 marker (associated to suppressive function) was used to define 2 different populations of Treg cells: CD39+ and CD39- Treg cells.

3.- Activation: Using CD38 and HLA-DR as surrogate markers of activation. Three different subsets were considered: CD38+DR-, CD38+DR+ and CD38-DR+. The level of activation was evaluated in the different subsets defined by CD45RA and CCR7, as well as in Treg cells.

4.- Replicative senescence: Using CD57 and CD28 markers that define 4 unique subsets. Senescent cells were defined as CD28-CD57+ and non-senescent as CD28+CD57-. Levels of senescence were evaluated in the different subsets defined by CD45RA and CCR7, as well as in Treg cells.

5.- Exhaustion: Using PD1 as a surrogate marker of exhaustion. Levels of PD1 were evaluated in the different subsets defined by CD45RA and CCR7.

6.- Response to homeostatic cytoquines. Expression of CD127 (the receptor for the homeostatic cytoquine IL7) was evaluated in the different subsets defined by CD45RA and CCR7.

Using this methodological approach a total of 350 different T-cell subsets was measured.
