# Supplementary material for: Class-modeling analysis reveals T-cell homeostasis disturbances involved in loss of immune control in elite controllers
Source: BMC Med. 2018 Feb 28;16:30. doi: 10.1186/s12916-018-1026-6 (PMC5830067; doi:10.1186/s12916-018-1026-6)
Supplement: Supplementary file 4 — Partial least-squares–class modeling (PLS-CM). (DOC 33 kb) [file 12916_2018_1026_MOESM4_ESM.doc]

Additional file 4. Partial Least Squares (PLS) class-modelling (PLS-CM).

Partial least squares (PLS) regression is a technique that reduces the number of predictor variables to a smaller set of uncorrelated components and performs least squares regression on these components, instead of on the original data. PLS regression is especially useful when the predictor variables are highly collinear, or when there are far more predictor variables than observations and ordinary least-squares regression either produces coefficients with high standard errors or fails completely. In biomedical sciences, PLS class modelling has been mainly used in studies involving a dataset of variables much larger than the number of observations [1], such as genomic [2], proteomic [3] and metabolomic [4] studies.

In PLS, the observable (measured) predictor variables are grouped together to generate a minimum set of latent variables (LV), i.e. variables that are not directly observed (measured) but rather inferred (through a mathematical model) from other variables that are observed (directly measured). Each LV is a linear combination of the original set of observable variables. A clear advantage of using latent variables is that it reduces the dimensionality of data. The number of LV included in the PLS model is optimized to maximize the explained variance of X (predictor variables) and Y (response variable) components of the model.

The global procedure to build a class model includes three steps: 1) a PLS regression; 2) distribution of the PLS fitted response for each class; 3) class modeling using a hypothesis test.

In this study, PLS-CM was used as a tool for modeling the different groups or classes of subjects included in the study (EC patients versus HC for the first PLS model; and EC controls versus EC cases for the second PLS model), using the set of immunological variables (350 in total) as the predictor variables and the subject´s group as the response (predicted) categorical variable. Values of 0 and 1 were assigned to the response variable for the two different categories (EC patient=0, HC=1 for the first model; EC control=0, EC case=1 for the second model). In a first step, PLS was used to make a predictor variables selection and reduce the original 350 to a small number. The parameter employed, called variable influence on projection, VIP, summarizes the importance of the X-variables (predictor variables), and is a weighted sum of squares of the PLS weights, which takes into account the amount of explained Y-variance (variance of the response variable) in each dimension. VIP scores estimate the importance of each variable in the projection used in a PLS model. A variable with a VIP score close to or greater than 1 can be considered important in the given model. Variables with VIP scores significantly less than 1 are less important and might be good candidates for exclusion from the model. The procedure was as follows:

*1.- PLS regression*

A data matrix with n samples and 350 predictor variables was used to fit a first PLS model (PLS-1 for EC patients versus HC, n=50 samples; and PLS-2 for EC controls versus EC cases, n=36 samples) without any data preprocessing and a binary response variable which takes the value zero or one depending on the category to which the sample belongs. This let us choose a smaller number of predictor variables with VIP value higher than 1 and simplify the analysis without losing any information. The final PLS models were constructed following the next steps:

1. Obtain the number of latent variables that minimized the root mean squared error in cross-validation, RMSECV, obtained by venetian blinds procedure.
2. Remove those samples with standardized residual (in absolute value) greater than 2.5 and with *Q* and *T^2^* values significantly higher than a threshold at 99% confidence level.
3. Repeat steps (a) and (b) until there were no outliers.

*2.- Distribution of fitted PLS response*

A distribution for each category or class was fitted with values calculated by PLS: Average values for each class were different from 0 and 1, which are the values when the PLS model explains 100% of the response variable. In fact, the distance between the estimated average values (µ_2_-µ_1_) is the percentage of the variance of the response variable explained by the PLS model.

*3.- Class modelling and risk curve*

In order to decide if an unknown sample belongs to one or another category, a threshold value, tv, between 0 and 1 must be established. If the value estimated by PLS is higher than tv the sample is classified to belong to one category, for estimated values lower than tv the sample is classified to belong to the other category.

A model for one class (e.g. “EC patient”), is in fact the acceptation region for the null hypothesis H0: the sample belongs to “EC patient” class. Therefore, the evaluation of the quality of a class model is given by its sensitivity (i.e. proportion of the samples of the class that are correctly assigned), while specificity is the proportion of samples correctly rejected. Both parameters can be stated in the context of a hypothesis test, sensitivity is an estimation of (1−α)×100, where α is the probability of first type error (that is the pr{to reject H0/H0 is true}) and the specificity is (1−β)×100, where β is the pr{reject H0/H0 is false}.

The risk curve is the plot of *β* error *versus* *α* error. Under normality hypothesis the parametric estimation of both probabilities is optimal; otherwise a non-parametrical methodology would be used. It is clear that both probabilities change in opposite directions, that is, *α* decrease when *β* increase and vice versa in a specific way.

Software

PLS models have been done with the PLS Toolbox running under MATLAB 7.0 and the fitting of distributions of probability were obtained with STATGRAPHICS Centurion XVI. A homemade function programmed in MATLAB was used to calculate the probabilities α, β and the risk curve.

References

1. Li L. Dimension reduction for high-dimensional data. Methods Mol Biol 2010; 620:417-434.
2. Boulesteix AL, Strimmer K. Partial least squares: a versatile tool for the analysis of high-dimensional genomic data. Brief Bioinform 2007; 8:32-44.
3. Jorgensen KM, Hjelle SM, Oye OK, Puntervoll P, Reikvam H, Skavland J, et al. Untangling the intracellular signaling network in cancer - a strategy for data integration in acute myeloid leukaemia. J Proteomics. 2011 74:269-81.
4. Gromski PS, Muhamadali H, Ellis DI, Correa E, Turner ML, Goodacre R. A tutorial review: metabolomics and partial least squares-discriminant analysis--a marriage of convenience or a shotgun wedding. Anal Chim Acta 2015; 870:10-23.
