# Supplementary material for: Class-modeling analysis reveals T-cell homeostasis disturbances involved in loss of immune control in elite controllers
Source: BMC Med. 2018 Feb 28;16:30. doi: 10.1186/s12916-018-1026-6 (PMC5830067; doi:10.1186/s12916-018-1026-6)
Supplement: Supplementary file 5 — Immunological variables (in CD4 and CD8 T-cell subsets) with significant differences between EC patients and healthy subjects (HCs) (Mann–Whitney U test). (DOC 119 kb) [file 12916_2018_1026_MOESM5_ESM.doc]

Additional file 5. Immunological Variables (in CD4 and CD8 T-cell subsets) with significant differences between EC patients and healthy subjects (HC) (Mann-Whitney U test).

| **Subsets of CD4 T-cells** | | | |
| --- | --- | --- | --- |
| **Cell Subset** | **Biological process** | **p-value** | **Selected by PLS model** |
|  |  |  |  |
| CD45RA+CD27-CCR7+ subset | Maturation stage | 0.004 | No |
| CD45RA-CD27-CCR7- subset | Maturation stage | 0.034 | Yes |
| CD45RA-CCR7- subset | Maturation stage | 0.012 | Yes |
| CD45RA+CD27+CCR7+CD95+ (Tscm) subset | Maturation stage | 0.003 | No |
| CD127+ subset | Maturation stage | 0.004 | Yes |
| CD127+ subset of CD45RA+CCR7+ cells | Maturation stage | <0.0001 | Yes |
| CD127+ subset of CD45RA+CCR7- cells | Maturation stage | 0.002 | Yes |
| CD127+ subset of CD45RA-CCR7+ cells | Maturation stage | 0.001 | Yes |
| CD127+ subset of CD45RA-CCR7- cells | Maturation stage | 0.001 | Yes |
| CD38+HLADR- subset | Activation | 0.006 | Yes |
| CD38+HLADR- subset of CD45RA+CD27+CCR7+ cells | Activation | 0.001 | Yes |
| CD38+HLADR- subset of CD45RA+CD27-CCR7- cells | Activation | 0.001 | Yes |
| CD38+HLADR- subset of CD45RA-CD27+CCR7+ cells | Activation | 0.042 | No |
| CD38+HLADR+ subset of CD45RA-CD27+CCR7+ cells | Activation | 0.009 | No |
| CD38+HLADR+ subset of CD45RA+CD27-CCR7- cells | Activation | 0.016 | No |
| CD38+HLADR+ subset of CD45RA-CD27-CCR7+ cells | Activation | 0.047 | No |
| CD38-HLADR+ subset of CD45RA-CD27+CCR7- cells | Activation | 0.013 | No |
| CD38+HLADR+ subset of CD45RA-CD27+CCR7- cells | Activation | 0.023 | No |
| CD38+HLADR- subset of CD45RA+CCR7+ cells | Activation | 0.001 | Yes |
| CD38+HLADR+ subset of CD45RA+CCR7+ cells | Activation | 0.013 | No |
| CD38+HLADR+ subset of CD45RA+CCR7- cells | Activation | 0.012 | No |
| CD38+HLADR- subset of CD45RA+CD27+CCR7+CD31+ (RTE) cells | Activation | 0.001 | Yes |
| CD38+HLADR+ subset of CD45RA+CD27+CCR7+CD95+ (Tscm) cells | Activation | 0.017 | No |
| CD38+HLADR- subset of CD39-Treg cells | Activation | 0.031 | No |
| CD95+PD1- subset of CD45RA-CD27-CCR7+ cells | CD95 / PD1 expression | 0.006 | Yes |
| CD127-CD25+ (Treg) subset | Regulatory T cells | 0.001 | No |
| CD45RA+CCR7+ subset of Treg cells | Regulatory T cells | 0.047 | Yes |
| CD45RA-CCR7+ subset of Treg cells | Regulatory T cells | 0.008 | Yes |
| CD45RA-CCR7- subset of Treg cells | Regulatory T cells | 0.014 | Yes |
| CD45RA+CCR7+ subset of CD39+Treg cells | Regulatory T cells | 0.049 | No |
| CD45RA-CCR7+ subset of CD39+Treg cells | Regulatory T cells | <0.0001 | Yes |
| CD45RA-CCR7- subset of CD39+Treg cells | Regulatory T cells | 0.002 | Yes |
| CD45RA+CCR7+ subset of CD39-Treg cells | Regulatory T cells | 0.028 | Yes |
| CD45RA-CCR7+ subset of CD39-Treg cells | Regulatory T cells | 0.036 | No |
| CD45RA-CCR7- subset of CD39-Treg cells | Regulatory T cells | 0.029 | Yes |
| CD28-CD57- subset | Senescence | 0.001 | No |
| CD28-CD57- subset of CD45RA+CCR7+ cells | Senescence | <0.0001 | No |
| CD28+CD57- subset of CD45RA+CCR7+ cells | Senescence | <0.0001 | Yes |
| CD28-CD57- subset of CD45RA+CCR7- cells | Senescence | 0.040 | No |
| CD28-CD57- subset of CD45RA-CCR7+ cells | Senescence | <0.0001 | No |
| CD28+CD57+ subset of CD45RA-CCR7+ cells | Senescence | 0.015 | No |
| CD28-CD57- subset of Treg cells | Senescence | 0.021 | No |
| CD28+CD57- subset of Treg cells | Senescence | 0.004 | Yes |
| **Subsets of CD8 T-cells** | | | |
| **Cell Subset** | **Biological process** | **p-value** | **Selected by PLS model** |
| CD45RA+CD27+CCR7+ subset | Maturation stage | 0.002 | Yes |
| CD45RA-CD27-CCR7- subset | Maturation stage | 0.001 | Yes |
| CD45RA+CD27-CCR7+ subset | Maturation stage | 0.021 | No |
| CD45RA+CCR7+ subset | Maturation stage | 0.012 | Yes |
| CD45RA-CCR7- subset | Maturation stage | 0.001 | Yes |
| CD45RA+CD27+CCR7+CD31+ (RTE) subset | Maturation stage | 0.003 | Yes |
| CD45RA+CD27+CCR7+CD95+ (Tscm) subset | Maturation stage | 0.001 | No |
| CD38+HLADR+ subset | Activation | 0.008 | No |
| CD38+HLADR- subset of CD45RA+CD27+CCR7+ cells | Activation | 0.014 | Yes |
| CD38+HLADR- subset of CD45RA+CD27-CCR7- cells | Activation | 0.008 | Yes |
| CD38+HLADR+ subset of CD45RA+CD27-CCR7- cells | Activation | 0.007 | No |
| CD38+HLADR- subset of CD45RA-CD27-CCR7- cells | Activation | 0.002 | Yes |
| CD38+HLADR+ subset of CD45RA-CD27-CCR7- cells | Activation | 0.007 | No |
| CD38+HLADR- subset of CD45RA-CD27+CCR7- cells | Activation | 0.025 | No |
| CD38-HLADR+ subset of CD45RA-CD27+CCR7- cells | Activation | 0.037 | No |
| CD38+HLADR- subset of CD45RA+CCR7+ cells | Activation | 0.048 | No |
| CD38+HLADR- subset of CD45RA+CCR7- cells | Activation | 0.043 | Yes |
| CD38+HLADR+ subset of CD45RA+CCR7- cells | Activation | 0.044 | No |
| CD38+HLADR- subset of CD45RA-CCR7- cells | Activation | 0.049 | No |
| CD38+HLADR+ subset of CD45RA+CD27+CCR7+CD95+ (Tscm) cells | Activation | 0.023 | No |
| CD38+HLADR- subset of CD45RA+CD27+CCR7+CD31+ (RTE) cells | Activation | 0.015 | Yes |
| CD38+HLADR+ subset of Treg cells | Activation | 0.005 | No |
| CD38-HLADR+ subset of Treg cells | Activation | <0.0001 | No |
| CD95+PD1- subset of CD38+HLADR- cells | CD95 / PD1 expression | <0.0001 | Yes |
| CD95+PD1- subset of CD38+HLADR+ cells | CD95 / PD1 expression | 0.041 | Yes |
| CD95+PD1- subset of CD38-HLADR+ cells | CD95 / PD1 expression | 0.011 | Yes |
| CD28-CD57- subset | Senescence | <0.0001 | Yes |
| CD28+CD57- subset | Senescence | 0.027 | Yes |
| CD28-CD57- subset of CD45RA+CCR7+ cells | Senescence | <0.0001 | Yes |
| CD28+CD57- subset of CD45RA+CCR7+ cells | Senescence | <0.0001 | Yes |
| CD28-CD57- subset of CD45RA+CCR7- cells | Senescence | 0.007 | No |
| CD28-CD57- subset of CD45RA-CCR7+ cells | Senescence | <0.0001 | No |
| CD28+CD57- subset of CD45RA-CCR7+ cells | Senescence | 0.035 | Yes |
| CD28-CD57- subset of CD45RA-CCR7- cells | Senescence | <0.0001 | Yes |
| CD28+CD57- subset of CD45RA-CCR7- cells | Senescence | 0.013 | Yes |
| CD28+CD57- subset of Treg cells | Senescence | 0.007 | Yes |
