# Supplementary material for: Class-modeling analysis reveals T-cell homeostasis disturbances involved in loss of immune control in elite controllers
Source: BMC Med. 2018 Feb 28;16:30. doi: 10.1186/s12916-018-1026-6 (PMC5830067; doi:10.1186/s12916-018-1026-6)
Supplement: Supplementary file 6 — Scatterplot showing loading values (coefficients) for the 77 immunological variables selected by the PLS model versus p values obtained when comparing levels of these variables between EC cases and EC controls with the Mann–Whitney U test. The dotted line marks the threshold for statistical significance (p < 0.05). (DOC 67 kb) [file 12916_2018_1026_MOESM6_ESM.doc]

Additional file 6. Scatter-plot graph showing loading values (coefficients) for the 77 immunological variables selected by PLS model versus p-values obtained when comparing levels of these variables between EC cases and EC controls by Mann-Whitney U test. Dotted line marks the treshold for statistical significance (p<0.05)
