# Supplementary material for: Class-modeling analysis reveals T-cell homeostasis disturbances involved in loss of immune control in elite controllers
Source: BMC Med. 2018 Feb 28;16:30. doi: 10.1186/s12916-018-1026-6 (PMC5830067; doi:10.1186/s12916-018-1026-6)
Supplement: Supplementary file 7 — Immunological variables (in CD4 and CD8 T-cell subsets) with significant differences between EC cases and EC controls (Mann–Whitney U test). (DOC 52 kb) [file 12916_2018_1026_MOESM7_ESM.doc]

Additional file 7. Immunological Variables (in CD4 and CD8 T-cell subsets) with significant differences between EC cases and EC controls (Mann-Whitney U test).

| **Subsets of CD4 T-cells** | | | |
| --- | --- | --- | --- |
| **Cell Subset** | **Biological process** | **p-value** | **Selected by PLS model** |
|  |  |  |  |
| CD38+HLADR- subset of CD45RA+CD27-CCR7- cells | Activation | 0.001 | Yes |
| CD38+HLADR- subset of CD45RA+CCR7- cells | Activation | 0.021 | Yes |
| CD38+HLADR- subset of CD45RA-CD27-CCR7+ cells | Activation | 0.049 | No |
| CD95-PD1+ subset | CD95 / PD1 expression | 0.012 | No |
| CD95-PD1+ subset of CD45RA-CD27+CCR7+ cells | CD95 / PD1 expression | 0.010 | No |
| CD95-PD1+ subset of CD45RA-CD27+CCR7- cells | CD95 / PD1 expression | 0.011 | No |
| CD95+PD1+ subset of CD45RA-CD27+CCR7+ cells | CD95 / PD1 expression | 0.041 | No |
| CD95+PD1+ subset of CD45RA+CD27-CCR7+ cells | CD95 / PD1 expression | 0.041 | No |
| **Subsets of CD8 T-cells** | | | |
| **Cell Subset** | **Biological process** | **p-value** | **Selected by PLS model** |
| CD45RA+CD27+CCR7+ subset | Maturation stage | 0.023 | Yes |
| CD45RA+CCR7+ subset | Maturation stage | 0.016 | Yes |
| CD45RA+CD27+CCR7+CD31+ (RTE) subset | Maturation stage | 0.027 | Yes |
| CD45RA+CD27-CCR7-subset | Maturation stage | 0.008 | Yes |
| CD45RA+CCR7-subset | Maturation stage | 0.013 | Yes |
| CD31+ subset | Maturation stage | 0.032 | Yes |
| CD95-PD1+ subset | CD95 / PD1 expression | 0.049 | No |
| CD95-PD1+ subset of CD45RA+CD27-CCR7+ | CD95 / PD1 expression | 0.03 | No |
| CD95+PD1- subset of CD45RA+CD27-CCR7- cells | CD95 / PD1 expression | 0.017 | Yes |
| CD28-CD57+ subset | Senescence | 0.017 | Yes |
| CD28-CD57+ subset of CD45RA+CCR7+ cells | Senescence | 0.03 | No |
| CD28+CD57- subset | Senescence | 0.03 | Yes |
| CD45RA-CCR7+ subset of Treg cells | Regulatory T cells | 0.048 | Yes |
